# Differential expression of lncRNAs during the HIV replication cycle: an underestimated layer in the HIV-host interplay

**DOI:** 10.1038/srep36111

**Published:** 2016-10-26

**Authors:** Wim Trypsteen, Pejman Mohammadi, Clarissa Van Hecke, Pieter Mestdagh, Steve Lefever, Yvan Saeys, Pieter De Bleser, Jo Vandesompele, Angela Ciuffi, Linos Vandekerckhove, Ward De Spiegelaere

**Affiliations:** 1 Department of Internal Medicine, HIV Cure Research Centre, Ghent University, Ghent, Belgium; 2 Institute of Microbiology (IMUL), Lausanne University Hospital and University of Lausanne, Lausanne, Switzerland; 3 Center Medical Genetics, Ghent University, Belgium; 4 Inflammation Research Center, Flanders Institute of Biotechnology (VIB), Ghent, Belgium; 5 Department of Biomedical Molecular Biology Ghent University, Ghent, Belgium; 6 Department of Respiratory Medicine, Ghent University, Ghent, Belgium; 7 Department of Morphology, Ghent University, Belgium

## Abstract

Studying the effects of HIV infection on the host transcriptome has typically focused on protein-coding genes. However, recent advances in the field of RNA sequencing revealed that long non-coding RNAs (lncRNAs) add an extensive additional layer to the cell’s molecular network. Here, we performed transcriptome profiling throughout a primary HIV infection *in vitro* to investigate lncRNA expression at the different HIV replication cycle processes (reverse transcription, integration and particle production). Subsequently, guilt-by-association, transcription factor and co-expression analysis were performed to infer biological roles for the lncRNAs identified in the HIV-host interplay. Many lncRNAs were suggested to play a role in mechanisms relying on proteasomal and ubiquitination pathways, apoptosis, DNA damage responses and cell cycle regulation. Through transcription factor binding analysis, we found that lncRNAs display a distinct transcriptional regulation profile as compared to protein coding mRNAs, suggesting that mRNAs and lncRNAs are independently modulated. In addition, we identified five differentially expressed lncRNA-mRNA pairs with mRNA involvement in HIV pathogenesis with possible *cis* regulatory lncRNAs that control nearby mRNA expression and function. Altogether, the present study demonstrates that lncRNAs add a new dimension to the HIV-host interplay and should be further investigated as they may represent targets for controlling HIV replication.

The interplay between the human immunodeficiency virus (HIV) and the host’s cellular defenses has been broadly studied to elucidate the underlying viral and antiviral molecular mechanisms. These studies typically focused on protein-coding genes and successfully identified several host factors involved in HIV pathogenesis and intracellular defense^1^,^2^,^3^,^4^,^5^,^6^. However, over the past decade it has become evident from genome-wide tiling arrays and RNA sequencing studies that the human genome is pervasively transcribed and that the majority of these transcribed genomic sequences are associated with non-coding RNAs (ncRNAs) rather than protein coding RNAs, underpinning their widespread presence in the cellular environment and adding a new layer of complexity to the cell’s molecular network^7^,^8^,^9^,^10^.

The class of ncRNAs is subdivided by length into small (<200 nt) and long (>200 nt) non-coding RNAs (lncRNAs). The lncRNAs constitute the bulk of ncRNAs with current databases describing up to ~62 000 long non-coding RNA genes compared to the ~20 000 known protein coding genes^11^,^12^. LncRNAs form a diverse group of molecules that can bind to DNA, RNA or proteins and are mainly involved in the regulation of gene expression, chromatin organization, nucleus-to-cytoplasm trafficking, RNA maturation and protein synthesis^9^. In addition, lncRNAs are frequently co-expressed with neighboring genes and show nuclear and/or cytoplasmic localization^8^,^13^. Furthermore, lncRNAs are crucial for normal cellular function and their dysregulation has already been linked to human diseases such as different types of cancer, autoimmune and neurodegenerative diseases^14^.

The ability of lncRNAs to control transcriptional processes offers unique possibilities for pathogens like HIV to hijack the cellular machinery and reshape gene expression in their favor^15^,^16^. Nonetheless, most attention to the involvement of ncRNAs in HIV-host interactions has been focused on small ncRNAs such as microRNAs (reviewed in refs 15 and 17). Only a few studies have aimed their attention at lncRNAs, resulting in the fact that the majority of these possibly relevant molecules remain unidentified^18^. To date, only two host lncRNAs and one HIV-encoded lncRNA are functionally characterized in the context of HIV infection or replication: *NEAT1, NRON* and the viral *ASP-L*^19^,^20^,^21^,^22^. Here, we present a focused extension study of a unique HIV time course experiment and specifically investigate lncRNA expression in the context of an HIV infection to shed light on the HIV-lncRNA regulatory landscape^23^.

## Materials and Methods

### HIV time course experiment

Two HIV time course experiments were conducted as previously described^23^. In short, (72 × 5 × 10^6) SupT1 cells were either mock-infected or infected with 15 μg p24 equivalent of an HIV-based vector (NL4.3Δenv::eGFP, VSV.G pseudotyped) by spinoculation at 1500 g for 30 min at room temperature in presence of 5 μg/ml polybrene (Sigma). After spinoculation, cells were washed and refreshed with culture medium (R-10). Subsequently, every two hours post infection, a fraction (3 × 10^6) of mock infected and infected SupT1 cells were washed with PBS and collected together with supernatant, until the 30 hour time point was reached.

### Assays for HIV life cycle markers

#### Measuring HIV reverse transcription levels

DNA was extracted with the DNeasy Blood and Tissue kit (Qiagen) according to the manufacturer’s instructions and DNA concentrations were measured with NanoDrop ND-1000 (NanoDrop). Early and late HIV reverse transcription products were quantified by real-time PCR as described before^23^. All real-time PCR reactions were performed in triplicate on the StepOnePlus Real-Time PCR system (Applied Biosystems) using standard cycling conditions, i.e. 2′ at 50 °C, 10′ at 95 °C, 40 cycles of 15″ at 95 °C and 1′ at 60 °C. Relative quantification was assessed by comparative Cq method and the fold changes were calculated by the ΔΔCq method, with the 24 hour time point as a reference. HMBS (PGBD) was used as an endogenous control (Supplementary Data 1).

#### Measuring HIV integration

For the quantification of integrated HIV DNA, first an Alu-gag PCR was carried out and subsequently a qPCR as described above. Alu-gag PCR: using 20 ng DNA, 0.4 mM primers (end concentration) (Supplementary Data 1), and Accuprime Pfx Supermix (Life Technologies) in a 25 μl final volume reaction. PCR cycling conditions were 5′ at 95 °C, followed by 25 cycles of 3″ at 95 °C, 15″ at 55 °C, 4′ at 68 °C, and finally 10′ at 68 °C. One tenth of this first PCR was used for qPCR as described above.

#### Measuring HIV transcription and particle release

Viral particle release was measured in the collected supernatant by p24 ELISA (Abbott Murex). Viral transcription/infection rates were assessed by GFP expression measurement by FACS analysis (FACS Calibur, Becton Dickinson).

### Modelling HIV markers

All markers were rescaled to the 24 hour time point and calculated as previously described^23^. Graphical output was generated using the R package *ggplot2* (v1.0.0) and the RStudio statistical software environment (v0.98.1028)^24^.

### Selection and quality control of samples for transcriptome analysis

In the two time course experiments, mock-infected and infected samples were selected at each of the following four time points, capturing different viral processes: early infection (2 h), peak of reverse transcription (6 h), peak of integration (18 h), viral release (30 h). This resulted in 16 samples. Total RNA was extracted using Trizol Reagent (Invitrogen) and RNA quality of all samples was assessed by Experion analysis (BioRad).

### Microarray analysis

The 16 selected samples were analyzed by a custom one-color microarray (8×60 k microarray, Agilent-050524 Human V2.0, GPL21113) that incorporates 40 k probes for lncRNA transcripts and 20 k probes for mRNA transcripts (microarray IDs: US45103088_255052410015, US45103088_255052410016). Sample preparation was performed with the Quick amp WT labelling kit (Agilent) according to the manufacturer’s instructions. In short, 100 ng total RNA was used per sample as input for the generation of fluorescent complementary RNA (cRNA). This involved cDNA synthesis and amplification with simultaneous incorporation of cyanine 3-labeled CTP by the T7 RNA polymerase. Subsequently, the cRNA was purified with the RNeasy kit (Qiagen) as instructed by the manufacturer’s protocol and RNA concentrations measured by NanoDrop ND-1000 (Thermo Scientific). Next, 600 ng of each cRNA sample was fragmented at 60 °C for 30 min and hybridized to the microarray slides for 17 hours in a hybridization oven rotator (Agilent) at 65 °C. At last, microarray slides were washed three times with gene expression washing buffers (Agilent) containing 0.005% Triton X-102 and scanned with the SureScan Microarray Scanner (Agilent) using one color scan settings: AgilentG3_GX_1Color.

### Microarray data processing and differential expression analysis

Raw microarray data is publicly available at the NCBI Gene Expression Omnibus (series GSE74818, http://www.ncbi.nlm.nih.gov/geo/query/acc.cgi?acc=GSE74818) and can be browsed online using the R2 data analysis tool (http://r2.amc.nl, dataset: Exp T cell lymphocyte Infection (lnc and coding) - Trypsteen). Processing of the raw fluorescent microarray signals and differential expression analysis were performed with the Bioconductor R package *limma* (v3.20.9)^25^. First, background correction and quantile normalization was applied in order to make the 16 arrays comparable. Next, control probes and low expressed probes were filtered out. Probes were considered expressed when the fluorescent signal was 50% brighter than the 95% quantile of the negative control probes on at least two arrays. Subsequently, a linear model was fitted for each gene to estimate the variability in the data and differential expression analysis was performed at each of the four selected time points. Fold changes were calculated by an empirical Bayes method and the returned p-values were corrected for multiple hypothesis testing by the Benjamini-Hochberg method^26^. Probes with adjusted p-values <0.05 were considered significantly differentially expressed and annotations were added to identify the gene symbols that are linked to the probes. Information on the different lncRNA classes was exported from LNCipedia (www.lncipedia.org), these included: intergenic, sense-overlapping, antisense, intronic and bidirectional lncRNA classes. In a last step, enhancer regions (eRNAs) were filtered by making use of the database of predicted human enhancers (DENdb, http://www.cbrc.kaust.edu.sa/dendb/index.php)^27^.

### qPCR validation of selected lncRNAs

cDNA synthesis on total RNA samples was performed with iScript (Bio-Rad) according to manufacturer’s protocol. Primers were designed with the IDT Primer Quest tool, primer specificity checked by NCBI BLAST analysis and secondary structures were determined with the mFold tool^28^. Primer efficiencies were tested on a standard curve of peripheral blood mononuclear cells. Next, primers were checked for specific amplification of the amplicon by smelting curve analysis and further used when amplification efficiency was observed between 90–110%^29^. All qPCR reactions were performed with the LightCycler 480 II (Roche) and LightCycler480 master mix (Roche). Relative quantification was performed by making use of reference gene normalization and the ΔΔCq method. Primers for following reference genes and lncRNAs were used: ACTB, GAPDH, UBC, lnc-BHLHE41-2, lnc-GSDMC-1, lnc-TRDMT1-1, lnc-LEF1-3, lnc-COX10-4, lnc-C7ORF44-1, lnc-ZBTB20-1, lnc-PABPN1L-1, lnc-GLB1L2-4, lnc-GKN2-1, lnc-LTBP3-1, lnc-AMZ2-1, lnc-ARRDC3-1, lnc-SYF2-1 and lnc-EXT1-1 (Supplementary Data 2, lncRNA gene names according to LNCipedia (www.lncipedia.org)^11^).

### Guilt-by-association analysis

Guilt-by-association analysis was performed as described previously^36^. In short, normalized gene expression values of the 16 samples were used to build a correlation matrix with the differentially expressed lncRNA and all expressed mRNAs. For each lncRNA, mRNAs are ranked according to their correlation coefficient and gene set enrichment analysis (GSEA) was performed using pathways from the Biocarta database^37^. A lncRNA was considered linked to a pathway in case of a GSEA False Discovery Rate (FDR)-value < 0.05. Visualization and network analysis was performed with Cytoscape using the built-in network analyzer tool^38^.

### Binding and expression target analysis (BETA): transcription factor analysis

Regulatory transcription factors (TFs) were determined for the differentially expressed mRNAs and lncRNAs at each time point through ‘binding and expression target analysis’ (BETA)^39^,^40^. In short, available Chip-seq data of 237 known transcription factors and the microarray gene expression data were integrated to identify putative active or repressive TFs and their target genes (cut-off used for enriched TF binding sites: 0.001). Next, clustering was performed by CLUTO software and visualization of TF networks was conducted with Cytoscape^38^,^41^.

### *Cis* and co-expression analysis of lncRNAs and neighboring mRNAs

Chromosomal coordinates based on the reference genome assembly GrCh37 were extracted from the LNCipedia database for the differentially expressed lncRNA transcripts^11^. Additionally, the Ensembl Biomart tool was used for the retrieval of chromosomal coordinates of all mRNA transcripts of the corresponding reference genome^30^. Based on the chromosomal coordinates, overlapping mRNAs and the closest neighboring mRNAs within the same topological associated domain (TAD) and a distance of up to 500 kb from transcription start and stop sites were determined for each differentially expressed lncRNA using a custom R script. Six categories were used to classify neighboring genes as previously described: full or partially overlapping, head to head, tail to tail, nearby to head (sense and antisense strand), nearby to tail (sense and antisense strand) (Supplementary Data 3)^13^,^31^. TAD information was downloaded from the ENCODE webserver (https://www.encodeproject.org/comparative/chromatin/#Hi-C) and was made available by Dixon et al^32^. Next, HIV interactions for the neighboring mRNAs were determined by making use of the HIV Interaction Database^33^. In addition, the epigenetic context of HIV linked mRNA-lnRNA pairs was determined for H3K4Me^1^, H3K4Me^3^, H3K27Ac, H3K36Me^3^ together with cap analysis gene expression (CAGE) peaks by making use of the Integrative Genomics Viewer^34^. Lastly, the co-expression database Co-LncRNA (http://www.bio-bigdata.com/Co-LncRNA) was browsed to determine similar co-expression events that correspond with our dataset^35^.

## Results

### Transcriptome changes and lncRNA involvement during HIV infection

**Figure 1:**
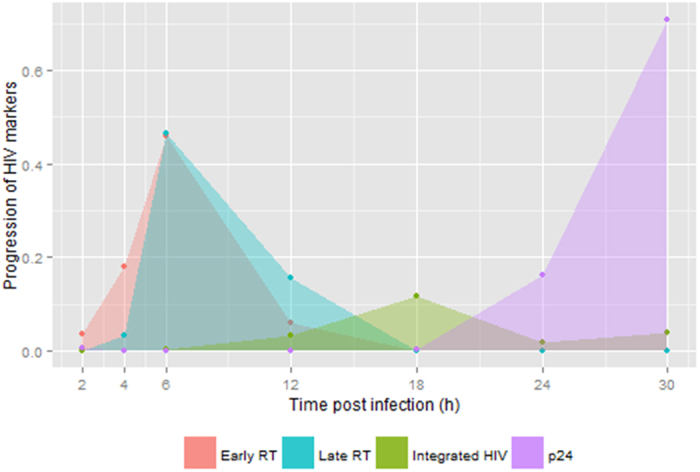
Progression of HIV replication cycle processes. The plot shows changes over time for viral measurements, including early reverse transcription (orange), late reverse transcription (cyan), integrated viral DNA (green) and virion-associated p24 protein expression (violet). Calculations for Fig. 1 are available in Supplementary Data 4E.

SupT1 cells were infected with a VSV-G pseudotyped HIV eGFP-based vector with ~70% success, as assessed by FACS analysis of GFP expression (Supplementary Data 4D). Different HIV replication cycle markers were measured over time post-infection: early and late reverse transcription products, integrated provirus and viral production products (Fig. 1 and Supplementary Data 4B and 4C). These assays showed peak activity respectively at 6, 18 and 30 hours post -infection consistent with previous reports and reflect the three main phases in the HIV life cycle: reverse transcription, integration and viral particle production (late phase)^23^. Subsequently, these three points were selected for further transcriptome analysis together with the 2-hour time point (Fig. 1 and Supplementary Data 4).

**Figure 2:**
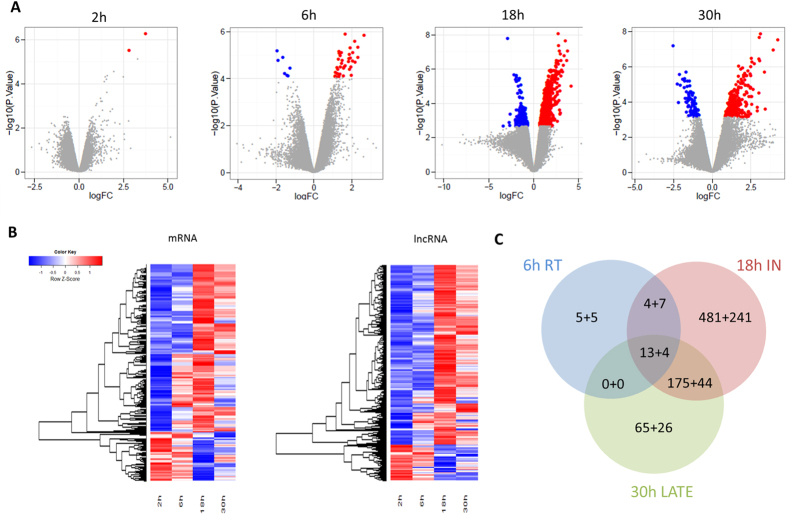
Overview of differential expression analysis between mock and HIV-infected cells at the four time points considered for transcriptome profiling. (**A**) Volcano plots showing all genes (grey dots). Significantly upregulated genes (red dots) and downregulated genes (blue dots) are highlighted for each time point. (**B**) Heatmaps of differentially expressed genes for the mRNA and lncRNA fractions. (**C**) Number of differentially expressed mRNAs and lncRNAs at peak of reverse transcription (RT), integration (IN) and viral particle release (LATE), respectively.

In the experimental system, 18 270 genes encompassing 12 281 mRNAs and 5989 lncRNAs were considered expressed using a selected threshold of 150% of the fluorescent background signal (Supplementary Data 5). In total, 1336 genes were found differentially expressed (DE) between mock and HIV-infected samples over all time points after correction for multiple hypothesis testing and a false discovery rate of 5%. These included 949 mRNAs and 387 lncRNAs, making that 29% of DE genes were lncRNAs with the majority of these DE lncRNAs originating from the intergenic or antisense class (Fig. 2, Table 1, Supplementary Data 6A and 6B). Differential expression gradually increased, with the majority of DE genes found at the 18-hour time point or at peak of HIV integration.

Fifteen out of 387 lncRNAs were selected for subsequent RT-qPCR validation based on high fold change and differential expression at more than one time point. RT-qPCR confirmed differential expression of 12 out of 15 lncRNAs identified by microarray transcriptome analysis (Supplementary Data 7). In addition, further validation of our DE genes included a comparison with the only previously published transcriptome dataset looking into mRNA and lncRNA expression upon HIV infection (Supplementary Data 8)^18^. Overall, we find 349 mRNAs and 25 lncRNAs that overlap between datasets when comparing similar time points (18 hpi and 30 hpi) with Peng’s 24 hpi. This corresponded with 44–45% of the mRNAs and 8–13% of the lncRNAs that overlap at the 18 h and 30 h time point in our dataset, respectively.

To evaluate and suggest functional roles for these 387 lncRNAs in HIV infection, three data analysis pipelines were used that integrate information of mRNAs and lncRNAs. First, a guilt-by-association analysis linked to gene set enrichment analysis (GSEA) was conducted to match lncRNAs to biological pathways. Second, a transcription factor analysis was performed to investigate the transcriptional regulation of lncRNA and mRNAs during the HIV replication cycle. Third, a co-expression analysis was performed looking at neighboring mRNA genes of differentially expressed lncRNAs.

### Identifying biological pathways associated with lncRNAs upon HIV infection

**Figure 3:**
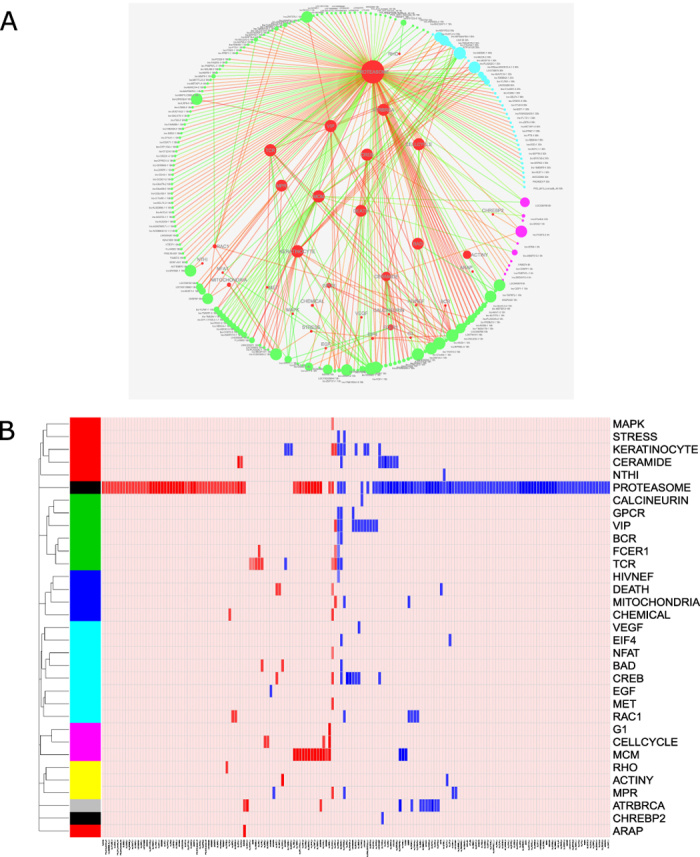
Overview of the guilt-by-association analysis. (**A**) Network representation of differentially expressed lncRNAs at the 6 h (purple dots), 18 h (green dots) and 30 h (blue dots) time point. lncRNAs are represented in the outer circle and connected pathways (red dots) at the inside of the circle. Connections show a negative association (red line) or a positive association (green line). The size of all circles is scaled to the number of connections. (**B**) Overview of lncRNA associations (columns) with 33 significantly enriched Biocarta pathways (rows). A negative association (red rectangles) and positive association (blue rectangles) are represented. Pathways are clustered according to the number of mutual genes.

Differentially expressed lncRNAs can be associated to biological pathways by applying the guilt-by-association principle with mRNAs. Therefore, expression profiles of all differentially expressed lncRNAs and all expressed mRNAs were integrated to identify associated pathways. This integrative analysis showed that 33 Biocarta pathways could be either negatively or positively associated to 173 DE lncRNA genes (p-value < 0.001) (Fig. 3).

The majority of differentially expressed lncRNAs are associated with the proteasome pathway and some smaller clusters can be found for apoptosis inhibition (VIP) and cell death, T-cell receptor signaling (TCR), Mini Chromosome Maintenance complex (MCM), genome integrity maintenance (BRCA1 and 2) and cell cycle progression (ATR), ceramide and keratinocyte pathways. Furthermore, one lncRNA, lnc-RPRML-3, is associated with the HIV Nef pathway and a few lncRNAs could be associated to multiple pathways, such as lnc-RPRML-3 (9 pathways), lnc-GSDMC-1 (10 pathways) and lnc-DNAJC8-1 (10 pathways).

### Transcriptional regulation of lncRNA and mRNA expression during HIV infection

**Figure 4:**
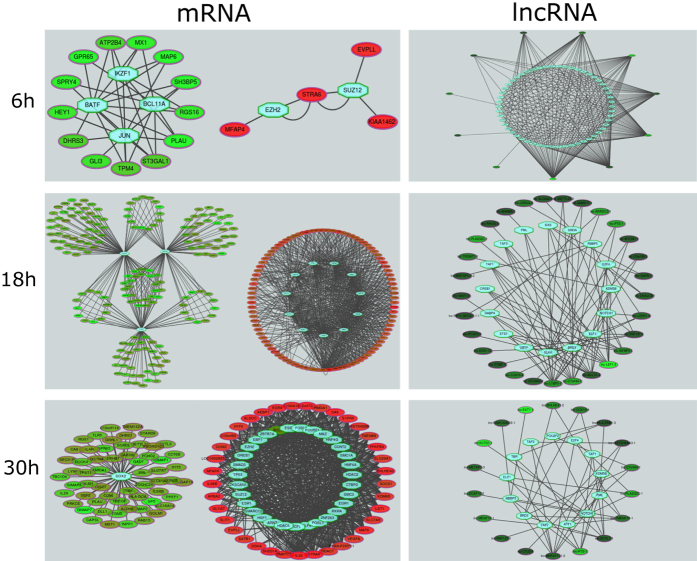
Transcriptional regulation for the mRNA and lncRNA fractions at the selected stages of the HIV replication cycle. Network representation for the upregulated (green) and downregulated (red) genes together with the enriched transcription factors (blue). Dark to bright color represents low to high fold change.

To shed light on lncRNA and mRNA transcriptional regulation, binding and expression target (BETA) analysis was performed for the differentially expressed mRNAs and lncRNAs at the different time points representing the three viral processes (Fig. 4).

For the mRNAs, transcription factor binding sites (TFBS) are found to be significantly enriched both in the downregulated and upregulated fraction whereas for lncRNA only enriched TFBS were found for upregulated lncRNAs. In addition, upregulated mRNAs are regulated by a select number of TFs whereas downregulated mRNAs are enriched for more TFs.

The upregulated lncRNAs are enriched for numerous TFBS (as compared to mRNAs) and some lncRNAs, i.e. lnc-LTBP3-1, show very large TF seed regions (>20 TFs), hinting at multifunctional lncRNAs that are inducible by many TFs.

Clustering analysis was performed to identify mRNAs and lncRNAs under similar transcriptional regulation for the 6 h, 18 h and 30 h time point (Supplementary Data 9A, 9B and 9C). However, data showed that lncRNA and mRNA have distinct transcriptional regulation profiles and small overlap in regulatory TFs, suggesting separate transcriptional control during HIV infection. In addition, most of the TFs found, have been linked to a role in HIV infection (Supplementary Data 10).

### HIV linked co-expression of mRNA and lncRNA upon HIV infection

Neighboring or close-by genes show different distances and orientations to each other and can be functionally linked and show correlated expression profiles, especially when situated in the same topological associated domain (TAD)^31^. This co-expression can be caused by regulation by the same (transcription) factor or by one gene regulating the expression of the other. In addition, lncRNAs are demonstrated to regulate protein coding gene expression at nearby (*cis* acting) or distant (*trans* acting) genomic loci^13^. Therefore, we explored the closest mRNAs of the differentially expressed lncRNAs and focused on HIV linked co-expression events (Supplementary Data 3).

For each differentially expressed lncRNA we determined the closest mRNAs within the same TAD and a genomic distance of 500 kb upstream or downstream of transcription start/stop site and found a total of 1162 lncRNA-mRNA pairs. Out of these, we identified a significant enrichment of 138 pairs (fisher’s exact test, p-value = 0.001) that contain a mRNA with previously described HIV interaction and 23 pairs that contain a mRNA that is also differentially expressed between mock and HIV samples (Supplementary Data 11, 12 and 13).

**Figure 5:**
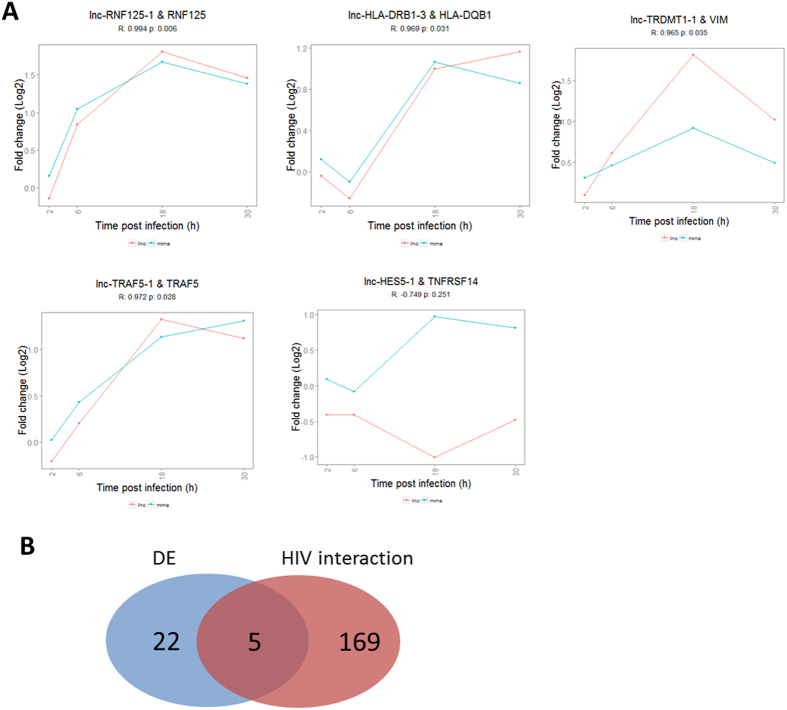
Overview of the co-expression analysis. (**A**) Expression profiles for the five HIV linked co-expression events, i.e. lncRNA (red) and mRNA (blue) pairs. (**B**) Number of differentially expressed pairs between mock and HIV-infected samples and HIV linked pairs.

To assess HIV linked co-expression events, expression profiles and epigenetic contexts were analyzed for five pairs that had a known mRNA-HIV interaction and were differentially expressed (Fig. 5 and Table 2, Supplemental Material 14). This resulted in significantly correlated expression profiles for four out of five pairs (Pearson correlation test, p-value < 0.05). In addition, one pair, lnc-HES5-1 & TNFRSF14, showed a negative expression correlation and opposite strand orientation, hinting at a possible *cis* regulatory lncRNA that induces transcriptional interference of the neighboring mRNA. Furthermore, the epigenetic context for this antisense lncRNA shows H3K36me^3^ along the transcribed region of the lncRNA and a and H3K4Me1 peak at the promotor site. In addition, 3 out of 5 of these co-expression events were also described in previous co-expression analysis reports. (Supplementary Data 15).

## Discussion

### Mapping the lncRNAome during the HIV replication cycle

lncRNA involvement in HIV infection has been poorly studied so far. Here, we explored modulation of the non-coding transcriptome throughout an HIV infection at selected time points representing different viral processes. This was performed with a VSVG pseudotyped HIV virus in order to acquire sufficient levels of infected cells to enable microarray based transcriptome analysis after a single round of infection and without the need of introducing antiretroviral drugs.

In total, 29% of differentially expressed genes between infected and non-infected cells were lncRNAs, showing that lncRNA involvement accounts for important and overlooked transcriptome changes during HIV infection. This is consistent with data from Peng *et al*. who found similar percentages of DE expressed ncRNAs in CD4+ T cells upon HIV infection at 12 h and 24 h post infection^18^. Furthermore, DE mRNAs and lncRNAs between our dataset and Peng *et al*. showed an overlap of 44–45% and 8–13%, respectively. A possible reason for the lower percentage of overlapping lncRNAs is that lncRNAs are found, an overage, to exert a shorter half-life than mRNAs and could change in a more time-dependent manner linked to specific cellular processes^42^. Hence, lncRNA expression might be influenced to a larger extent than mRNAs because the datasets differ in the time points considered for transcriptome profiling. Nonetheless, these overlapping mRNAs and lncRNAs validate our experimental setup and form ideal targets to be further investigated in the context of HIV infection. For instance, a well-known and characterized lncRNA, MALAT1 (lnc-SCYL1-1) is found in both datasets and is already linked to viral infection^43^. In addition, in HIV infected individuals MALAT1 is shown as a potential biomarker^44^. In this context, it would be worthwhile exploring and comparing transcriptome datasets of other viral infections (i.e. CMV, HSV) to pinpoint lncRNAs involved in the broad process of antiviral defense. Our dataset is publicly available and can contribute to this goal.

To date, only a few hundred lncRNAs are functionally validated^45^,^46^. This limits the possibility to associate biological meaning based on existing data analysis tools that are available for mRNAs. Therefore, we integrated three data analysis pipelines to associate biological functions to the differentially expressed lncRNAs.

### Potential roles of lncRNA in HIV replication

Virus-host interactions drive evolution with cycles of alternating countermeasures. In this context, HIV circumvents host’s cellular defenses rapidly and finds numerous escape mechanisms^47^. Interestingly, the guilt-by-association analysis showed that the majority of DE lncRNAs upon HIV infection could be functionally linked to the proteasome pathway. Indeed, it has been shown that (immuno-) proteasomes are recruited for cellular defense against incoming pathogens (i.e. HIV)^48^,^49^,^50^. These complexes digest viral proteins and provide small peptides that can be presented by Major Histocompatibility Complex I (MHC-I) molecules on the cell surface in order to be recognized and killed by other immune cells. On the other hand, HIV has evolved a way to counteract this by Nef-mediated downregulation of the (immuno-) proteasomes and MHC-I presentation^51^. Other examples of proteasomal countermeasures include the Vif-mediated ubiquitination of the HIV restriction factor APOBEC3G/F, flagging it for proteosomal degradation, as well as Vpu-mediated downregulation of CD4 and tetherin through proteosomal degradation^52^,^53^,^54^. In addition, the proteasome has been involved in the life cycle of many other viruses^48^,^55^. Therefore, we hypothesize that the proteasome plays an important role in HIV escape mechanisms but also in cellular defense against pathogens and that lncRNAs are widely involved in this interplay between virus and host.

A second group of lncRNAs was associated with apoptotic/cell death pathways. HIV relies on a fine balance between pro and anti-apoptotic signals to complete its life cycle. Infected cells should survive long enough to allow reverse transcription of HIV RNA to DNA, integration of the DNA in the host genome and production of new virions. On the other hand, the host cell can induce pro-apoptotic signals as a matter of defense and prevent the infection from spreading^56^. An example of such an association is provided by lnc-ARRDC3-1 (SCAL1) which is overexpressed in HIV infected cells and upregulated by the Nuclear factor erythroid 2-like 2 (NFE2L2, or Nrf2), a transcription factor regulating survival proteins in T-cells^57^. In addition, knockdown of SCAL1 induces cytotoxicity through an unknown pathway^58^. Our observation of SCAL1 overexpression in HIV infection supports that this lncRNA mediates cell survival in HIV infected T-cells.

Furthermore, several lncRNAs can be linked to the process of HIV integration. Viral integration causes DNA damage that is sensed by BRCA molecules that try to repair and cause cell cycle arrest before undergoing new DNA replication cycles^15^. Indeed, we identified lncRNAs linked to pathways involved in DNA repair (ATR/BRCA), cell cycle (CellCycle, G1) and DNA replication (MCM).

One lncRNA, lnc-RPRML-3, could be positively linked to a pathway involving HIV Nef-mediated apoptosis. Indeed, Nef upregulates the expression of FAS Ligand on the cell surface, thereby promoting killing of uninfected bystander CD4/CD8 T cells^59^,^60^. On the other hand, we found lncRNAs linked to the VIP (vasoactive intestinal peptide) pathway that is a host defense mechanism to inhibit FAS ligand expression^61^.

Here, lncRNAs show repeated involvement in the interplay between virus and host and emphasize that these pathways deserve further investigation to clarify their exact contribution.

### Transcriptional regulation of lncRNA and mRNA during HIV infection

The transcription factor analysis revealed that DE lncRNAs and mRNAs show distinct and separate transcriptional regulation profiles upon HIV infection. This finding is remarkable, as it suggests that lncRNAs and protein coding genes are controlled by different regulatory elements. Therefore, we can speculate that these transcriptional responses are independent from one another, provide transcriptional feedback loops or are part of integrated (viral/anti-viral) mechanisms guided by different TFs.

Furthermore, we were able to link mRNA and lncRNA expression to TFs with a characterized role in HIV infection and/or a physical interaction with an HIV protein. For instance, downregulation of mRNAs was mainly regulated by PRC2 complex members SUZ12, EZH2 and CTBP2. This complex was shown to be largely involved in HIV latency establishment and epigenetic silencing^62^. PRC2 is also linked to proteasome and ubiquitination pathways which corroborates guilt-by-association findings^63^. Next, we found multiple TFs that were described to directly interact with HIV Tat. Hence, we might speculate that the lncRNA targets of these TFs are Tat induced. For instance, TAF1/3/7 are represented at the 18 h and 30 h time point regulating lncRNA expression. These TAFs take part in the TFIID-complex (transcription factor IID) that is described to be directly interacting with HIV Tat and can guide RNAPOLII transcribed target genes^64^. Beside Tat linked TFs, we found TFs that guide lncRNA expression with a role in the regulation of HIV transcription. The TF CHD1 is a chromatin reassembly factor that acts as a positive regulator of HIV transcription as upon knockdown a total block of HIV transcription was previously observed^65^. ATF3 is part of the SWI/SNF complex and is responsible for shuttling this complex to the HIV promoter and guide HIV transcription. This complex is well characterized in HIV infection and important in HIV latency^66^,^67^.

LncRNAs were, on average, connected to more TFs as compared to mRNAs, indicating that specific lncRNAs can be induced by multiple TFs. In addition, some lncRNAs show very large TF seed regions and can be identified as hubs, inducible by many TFs, one such lncRNA was MALAT1 (lnc-SCYL1-1).

Lastly, all upregulated mRNAs at the 30 h time point displayed SOX2 regulation. In our dataset a lncRNA, SOXOT2, was differentially expressed (upregulated) at 30 h and spans the single exon gene SOX2, indicating possible lncRNA regulation of mRNA transcription. Indeed, SOXOT2 has been described as a regulating factor of SOX2 expression in breast cancer^68^.

### HIV linked co-expression of lncRNA and mRNA

*Cis* and co-expression analysis showed that expression profiles of the majority of the 23 differentially expressed lncRNA-mRNA pairs were correlated, suggesting that these lncRNAs are co-regulated and involved in the same biological process. Furthermore, it is possible that these lncRNAs regulate the expression of these mRNAs, as it has been shown for nearby lncRNAs to modulate mRNA expression levels^13^. However, functional validation of lncRNAs is needed to help understand their exact effects on nearby mRNAs and biological functions.

Interestingly, we identified five differentially expressed lncRNA-mRNA pairs with mRNA involvement in HIV pathogenesis (HIV linked co-expression). Amongst these correlated pairs we found RNF125, TRAF5 and VIM with a characterized role in HIV infection. RNF125 is an E3 ubiquitin ligase protein that promotes proteasomal degradation of RIG-I (retinoic acid-inducible gene 1), an innate immune sensor for viral RNA/DNA and activator of interferon type I, thereby reducing HIV transcription^69^. Therefore, we hypothesize that lnc-RNF125 may play a role in the RNF125-mediated downregulation of HIV replication. TRAF5 is a member of the TNF receptor associated factor family (TRAFs) and involved in HIV replication in monocyte derived macrophages as an interaction partner of HIV Nef. This viral protein mediates the activation of TRAF2, 5 and 6, thereby inducing NF-ƙB (nuclear factor kappa-light-chain-enhancer of activated B cells) release and transport to the nucleus with increased HIV-1 replication^70^. VIM (vimentin) is a major component of the cytoskeleton and can be cleaved by HIV-1 protease to alter the cytoskeleton for improved HIV-1 transport^71^. In addition, VIM is found incorporated in ribonuclear protein complexes that protect and keep HIV-1 transcripts in the cytoplasm, ready for viral particle formation^72^.

Although lcnRNAs have been implicated in mRNA regulation, testing specific lncRNA knockdown prior to HIV infection should help understand their effects on nearby mRNAs and biological functions.

## Conclusion

The present study provides a comprehensive map of differentially expressed lncRNAs throughout the HIV replication cycle and reveals a new and underestimated dimension in the HIV-host interplay. Furthermore, we introduced integrative analysis approaches to suggest biological roles for the DE lncRNAs in the context of HIV infection. Although our results are based on an *in vitro* cell line model for HIV infection with a pseudotyped HIV virus, these findings suggest that lncRNAs represent promising and potential targets for controlling HIV replication. These results need to be further explored and validated in primary cell models in order to fully grasp the impact of specific lncRNAs on HIV replication.

## Additional Information

**How to cite this article**: Trypsteen, W. *et al*. Differential expression of lncRNAs during the HIV replication cycle: an underestimated layer in the HIV-host interplay. *Sci. Rep.* **6**, 36111; doi: 10.1038/srep36111 (2016).

**Publisher’s note:** Springer Nature remains neutral with regard to jurisdictional claims in published maps and institutional affiliations.

## Supplementary Material

Supplementary Information

Supplementary Information

Supplementary Information

Supplementary Information

Supplementary Information

Supplementary Information

Supplementary Information

## Figures and Tables

**Table 1:** Overview of differential expression analysis at the gene level.

Differential expression (gene level)						
	2 hpi	6 hpi	18 hpi	30 hpi	Total	Total unique
mRNA	1	22	673	253	949	744
* Up*	1	17	571	201	790	
* Down*	0	5	102	52	159	
lncRNA	1	16	296	74	387	328
* Up*	1	16	216	56	289	
* Down*	0	0	80	18	98	
total	2	38	969	327	1336	1072

Number of differentially expressed genes for the four time points considered. If multiple probes were present per gene, one was used for counting the number of differentially expressed genes (Supplementary Data 6). hpi: hours post infection.

**Table 2:** Co-expression analysis. Overview of five differentially expressed lncRNA-mRNA pairs with HIV interaction.

nr	chr	LNCipedia gene ID	strand	HGNC	strand	distance	HIV interaction (HIVIntDb)
1	18	lnc-RNF125-1	+	RNF125	+	overlap	env, tat
2	6	lnc-HLA-DRB1-3	−	HLA-DQB1	−	overlap	env, nef, gag, tat, vif, vpu
3	1	lnc-TRAF5-1	+	TRAF5	+	7808	env, nef
4	10	lnc-TRDMT1-1	−	VIM	+	19	env, gag, gag-pol, vif, vpr
5	1	lnc-HES5-1	−	TNFRSF14	+	overlap	env, vif
