# Corrigendum: Differential expression of lncRNAs during the HIV replication cycle: an underestimated layer in the HIV-host interplay

**DOI:** 10.1038/srep41112

**Published:** 2017-01-24

**Authors:** Wim Trypsteen, Pejman Mohammadi, Clarissa Van Hecke, Pieter Mestdagh, Steve Lefever, Yvan Saeys, Pieter De Bleser, Jo Vandesompele, Angela Ciuffi, Linos Vandekerckhove, Ward De Spiegelaere

Scientific Reports 6: Article number: 3611110.1038/srep36111; published online: 10 26 2016; updated: 01 24 2017

In this Article, Yvan Saeys is incorrectly listed as being affiliated with ‘Department of Biomedical Molecular Biology Ghent University, Ghent, Belgium’. The correct affiliations are listed below:

Inflammation Research Center, Flanders Institute of Biotechnology (VIB), Ghent, Belgium.

Department of Respiratory Medicine, Ghent University, Ghent, Belgium.

In addition, Pieter De Bleser is incorrectly listed as being affiliated with ‘Department of Respiratory Medicine, Ghent University, Ghent, Belgium’. The correct affiliations are listed below:

Inflammation Research Center, Flanders Institute of Biotechnology (VIB), Ghent, Belgium.

Department of Biomedical Molecular Biology Ghent University, Ghent, Belgium.

