# Supplementary material for: Differential expression of lncRNAs during the HIV replication cycle: an underestimated layer in the HIV-host interplay
Source: Sci Rep. 2016 Oct 26;6:36111. doi: 10.1038/srep36111 (PMC5080576; doi:10.1038/srep36111)

Supplementary Data

Differential expression of lncRNAs during the HIV replication cycle: an underestimated layer in the HIV-host interplay

Authors: Trypsteen Wim[1], Mohammadi Pejman[2],Van Hecke Clarissa[1], Mestdagh Pieter[3], Lefever Steve[3], Saeys Yvan[4,5], De Bleser Pieter[4,6], Vandesompele Jo[3], Ciuffi Angela[2], Vandekerckhove Linos[1*+], De Spiegelaere Ward[1+]

(+) Equal contribution

Affiliations: [1] Department of Internal Medicine, HIV Cure Research Centre, Ghent University, Ghent, Belgium. [2] Institute of Microbiology (IMUL), Lausanne University Hospital and University of Lausanne, Lausanne, Switzerland. [3] Center Medical Genetics, Ghent University, Belgium. [4] Inflammation Research Center, Flanders Institute of Biotechnology (VIB), Ghent, Belgium. [5] Department of Biomedical Molecular Biology Ghent University, Ghent, Belgium. [6] Department of Respiratory Medicine, Ghent University, Ghent, Belgium.

Corresponding author: Vandekerckhove Linos; De Pintelaan 185, De Pintepark Building, Ghent University Hospital, 9000 Ghent, Belgium; tel: +3293323398, fax: +3293323895; linos.vandekerckhove@ugent.be

Supplementary Data 1: HIV life cycle primers and probes

Supplementary Data 2: qPCR primers lncRNA validation

Supplementary Data 3: Approach neighboring gene analysis for lncRNAs

Supplementary Data 4: Overview of all viral assays (dataset)

Supplementary Data 5: Expression values of mRNA and lncRNAs (dataset)

Supplementary Data 6: Differential expression of mRNA and lncRNAs. A: dataset and B: graphical overview

Supplementary Data 7: qPCR validation of 15 selected differentially expressed lncRNAs

Supplementary Data 8: Overlap differential expression Peng et al. (dataset)

Supplementary Data 9: Transcription factor analysis results in clusters. A: 6hpi, B: 18hpi, C: 30hpi

Supplementary Data 10: Enriched transcription factors for mRNA and lncRNAs with an HIV link (dataset)

Supplementary Data 11: Neighbouring gene (cis) analysis: all lncRNA-mRNA pairs (dataset)

Supplementary Data 12: HIV enrichment analysis in lncRNA-mRNA pairs

Supplementary Data 13: Expression profiles of the 23 differentially expressed lncRNA-mRNA pairs upon HIV infection

Supplementary Data 14: Epigenetic context of the 5 HIV linked lncRNA-mRNA pairs

Supplementary Data 15: Previously described coexpression profiles of the HIV linked lncRNA-mRNA pairs

Supplemental Data 1. Overview of the primers and probes used for the HIV life cycle markers.

HIV product	HIV process	Gene Symbol	Direction	5'-3' sequence
U5 (560-579)	HIV early RT	-	forward	GTGCCCGTCTGTTGTGTGAC
U5-PBS (642-623)	HIV early RT	-	reverse	GGCGCCACTGCTAGAGATTT
U5 (588-621)	HIV early RT	-	FAM-TAMRA probe	CTAGAGATCCCTCAGACCCTTTTAGTCAGTGTGG
U5 (557-576)	HIV late RT	-	forward	TGTGTGCCCGTCTGTTGTGT
psi (699-680)	HIV late RT	-	reverse	GAGTCCTGCGTCGAGAGATC
PBS (633-652)	HIV late RT	-	FAM-TAMRA probe	CAGTGGCGCCCGAACAGGGA
-	integrated, first PCR	Alu (host)	forward	GCCTCCCAAAGTGCTGGGATTACA
gag (803-782)	integrated, first PCR	-	reverse	GCTCTCGCACCCATCTCTCTCC
R (522-543)	integrated, nested PCR	-	forward	GCCTCAATAAAGCTTGCCTTGA
U5 (622-599)	integrated, nested PCR	-	reverse	TCCACACTGACTAAAAGGGTCTGA
U5 (563-591)	integrated, nested PCR	-	FAM-TAMRA probe	CCCGTCTGTTGTGTGACTCTGGTAACTAG
-	-	HMBS (PBGD) (GeneID:3145)	forward	AAGGGATTCACTCAGGCTCTTTC
-	-	HMBS (PBGD) (GeneID:3145)	reverse	GGCATGTTCAAGCTCCTTGG
-	-	HMBS (PBGD) (GeneID:3145)	VIC-MGB_NFQ probe	CCGGCAGATTGGAGAGAAAAGCCTGT

Supplemental Data 2. Overview of the qPCR primers used for three reference genes and 15 lncRNAs.

Gene Symbol	Accession number	lncipedia TranscriptID	Direction	Sequence
GAPDH	NM_002046.4	-	forward	5’-AGCCTCAAGATCATCAGCAATGCC-3’
			reverse	5’-TGTGGTCATGAGTCCTTCCACGAT-3’
ACTB	NM_001101.3	-	forward	5’-TTCCTTCCTGGGCATGGAGT-3’
			reverse	5’-TACAGGTCTTTGCGGATGTC-3’
UBC	NM_021009.5	-	forward	5’-TTGGGTCGCAGTTCTTGTTTGTGG-3’
			reverse	5’-TGACATTCTCGATGGTGTCACTGG-3’
-	-	lnc-C7orf44-1:1	forward	5’-ATGGCAACTCTGTGGGAATC-3’
			reverse	5’-CCCTGCTAGTCCATCCAGAA-3’
-	-	lnc-COX10-4:1	forward	5’-GGGAGGTTGGGCTTACTTTT-3’
			reverse	5’-ACATGGCAATGTCAATGGAA-3’
-	-	lnc-PABPN1L-1:1	forward	5’-ACTTCCACAGGGGCCAGAC-3’
			reverse	5’-AGGACGGTCCCAAATAGCAT-3’
-	-	lnc-GKN2-1:1	forward	5’-AGATGACAAGAACCTGGATTCAC-3’
			reverse	5’-ACAAGCAGCGGCCTTCTAAC-3’
-	-	lnc-LTBP3-1:1	forward	5’-CTCAGTGCGCCGACTATGT-3’
			reverse	5’-TCCTGCTTATGTGGGGATATG-3’
-	-	lnc-AMZ2-1:2	forward	5’-AGTGAGGCAGGGATGTCAGT-3’
			reverse	5’-AATCTCAGGGCACAGCAAGT-3’
-	-	lnc-LEF1-3:2	forward	5’-ACATCTCTTCAGCCCAGCAC-3’
			reverse	5’-TGCCACATTCATTCAACTGC-3’
-	-	lnc-ALDH1A2-4:1	forward	5’-GGCAGAAAGAGGTCCCTGTA-3’
			reverse	5’-ATTTTGAGCCAGTTGGGTTT-3’
-	-	lnc-ARRDC3-1:1	forward	5’-TGACTAAAACAATGGGGCATTT-3’
			reverse	5’-GATGGCCCAACCTACAGAGA-3’
-	-	lnc-ZBTB20-1:1	forward	5’-CAAGGTTTCTGTTTTGTGTGGA-3’
			reverse	5’-TTCCTTCATATTCAAACTTCACAA-3’
-	-	lnc-SYF2-1:1	forward	5’-ACCACCGTTGGAGGAAACTC-3’
			reverse	5’-TAATCCACAAGCCCAAGGTC-3’

Gene Symbol	Accession number	lncipedia TranscriptID	Direction	Sequence
-	-	lnc-TRDMT1-1:1	forward	5’-TGAAGCATTTGGATTACCAAGA-3’
			reverse	5’-GAGTCAGCACTAAAAAGGCAGA-3’
-	-	lnc-EXT1-1:1	forward	5’-TGCTTTCCTCTGTGTTAGAGCA-3’
			reverse	5’-AGCCACCTCTACAGCCATGT-3’
-	-	lnc-GSDMC-1:2	forward	5’-TGCAGGTTCACTGGAAATGA-3’
			reverse	5’-AGCGAAGAGTGTGGAGAACA-3’
-	-	lnc-BHLHE41-2:10	forward	5’-TATGCCAACTCCACCTTTGC-3’
			reverse	5’-GCTCACAGTTCTGCAGGCTTA-3’
-	-	lnc-GLB1L2-4:1	forward	5’-CCACTGGCAATCAGAGTTGTT-3’
			reverse	5’-CAGAGGCAGGTCTGCAAGAG-3’

Supplemental Data 3. Overview of *cis* analysis for sense and antisense lncRNA transcripts with the 6 categories used for nearby mRNAs (range of 500 kb).

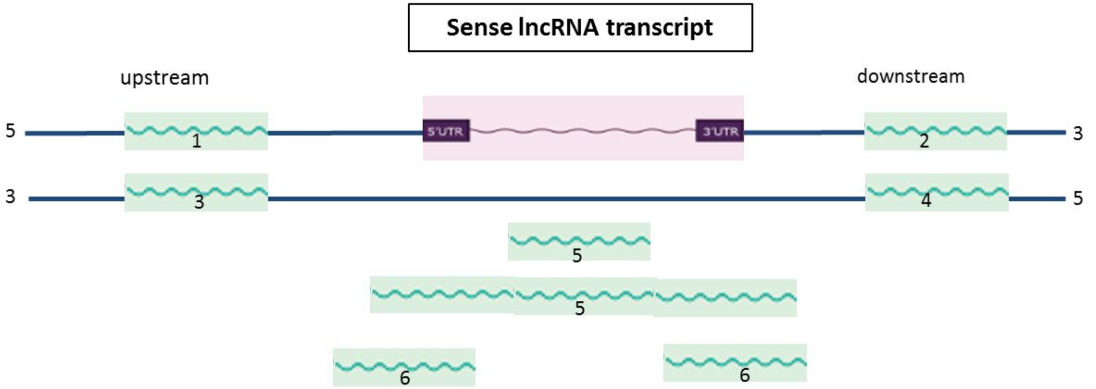

1) upstream sense: Tail-Head
2) downstream sense: Tail-Head

3) upstream antisense: nearby Head
4) downstream antisense: nearby Tail

5) full overlap
6) partial overlap

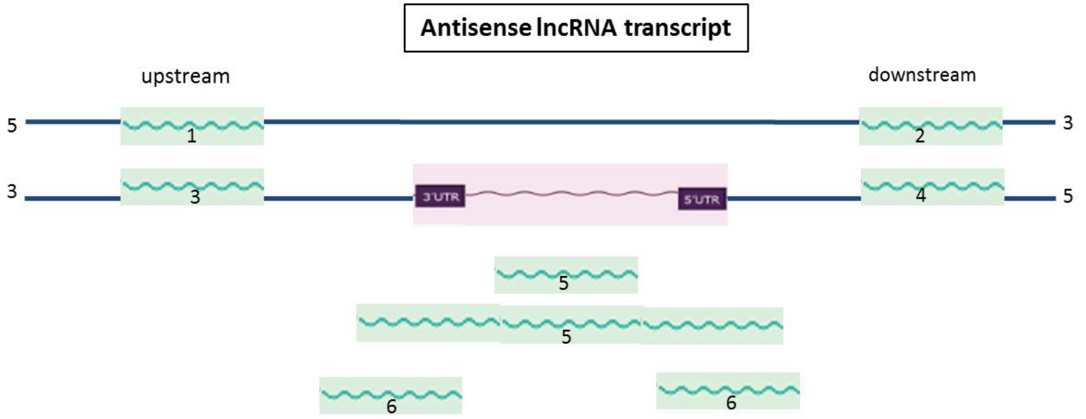

1) upstream sense: nearby Tail
2) downstream sense: nearby Head

3) upstream antisense: Tail-Head
4) downstream antisense: Tail-Head

5) full overlap
6) partial overlap

Supplementary Data 6B. Overview total DE genes with lncrna classes

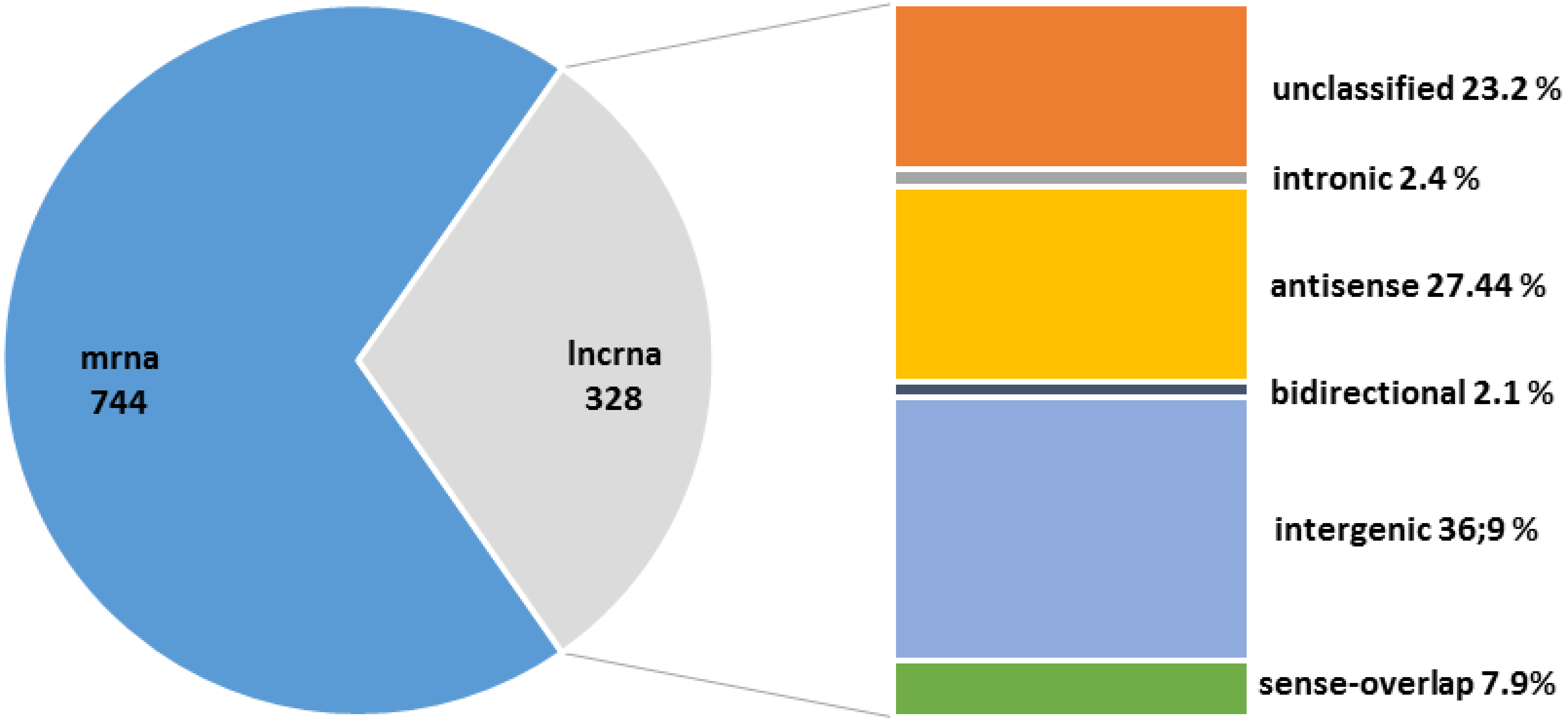

Supplemental Data 7: qPCR validation of the microarray analysis for 15 lncRNAs. Differential expression between mock and HIV-infected cells is represented by log_2(fold change) values of qPCR (red bars) and microarray (green bars). lncRNAs that were not validated are shown in grey.

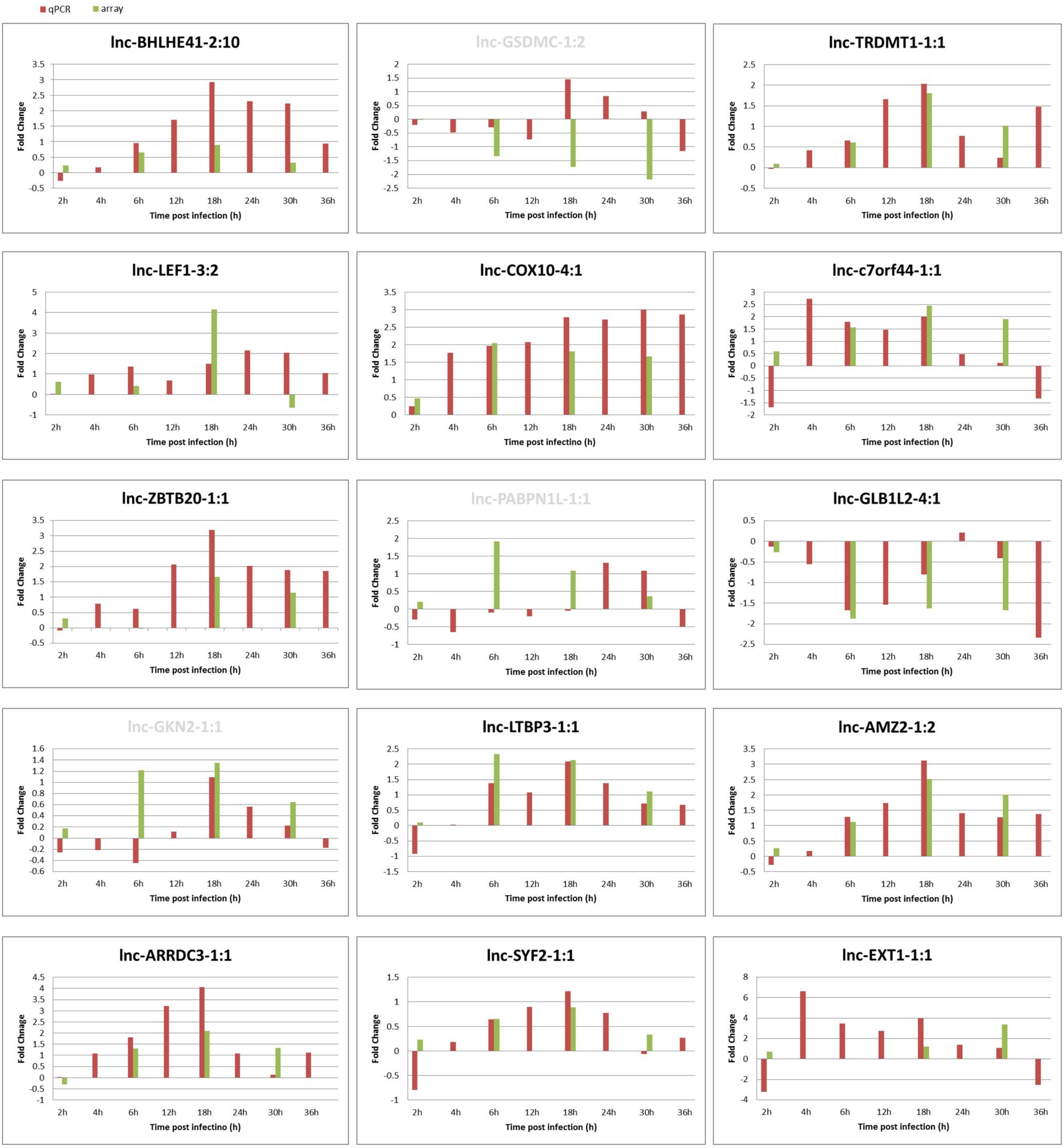

Supplementary Data 9. Transcription factor analysis results (clustered). A: 6hpi, B: 18hpi, C: 30hpi

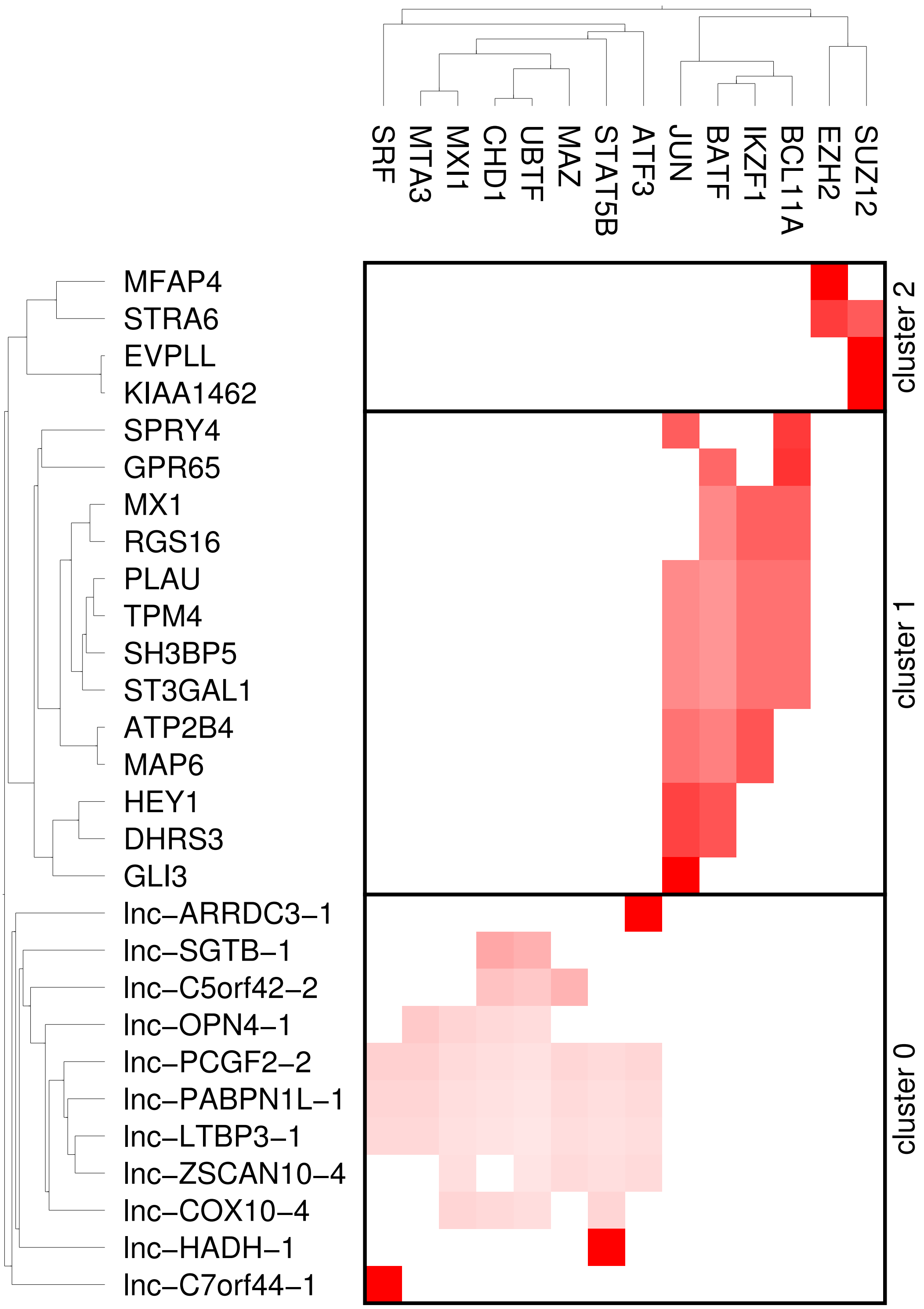
SRF
MTA3
MXI1
CHD1
UBTF
MAZ
STAT5B
ATF3
JUN
BATF
IKZF1
BCL11A
EZH2
SUZ12
MFAP4
STRA6
EVPLL
KIAA1462
SPRY4
GPR65
MX1
RGS16
PLAU
TPM4
SH3BP5
ST3GAL1
ATP2B4
MAP6
HEY1
DHRS3
GLI3
lnc–ARRDC3–1
lnc–SGTB–1
lnc–C5orf42–2
lnc–OPN4–1
lnc–PCGF2–2
lnc–PABPN1L–1
lnc–LTBP3–1
lnc–ZSCAN10–4
lnc–COX10–4
lnc–HADH–1
lnc–C7orf44–1
cluster 2
cluster 1
cluster 0

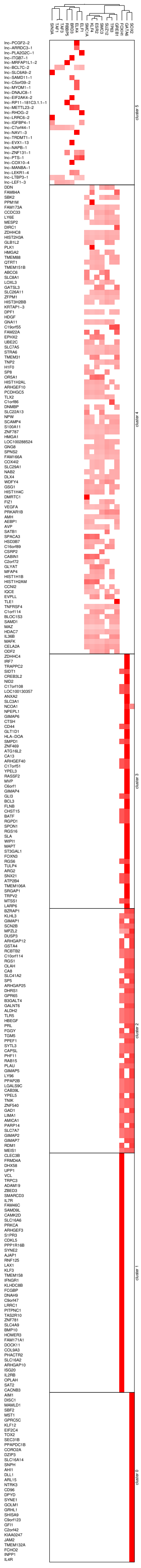

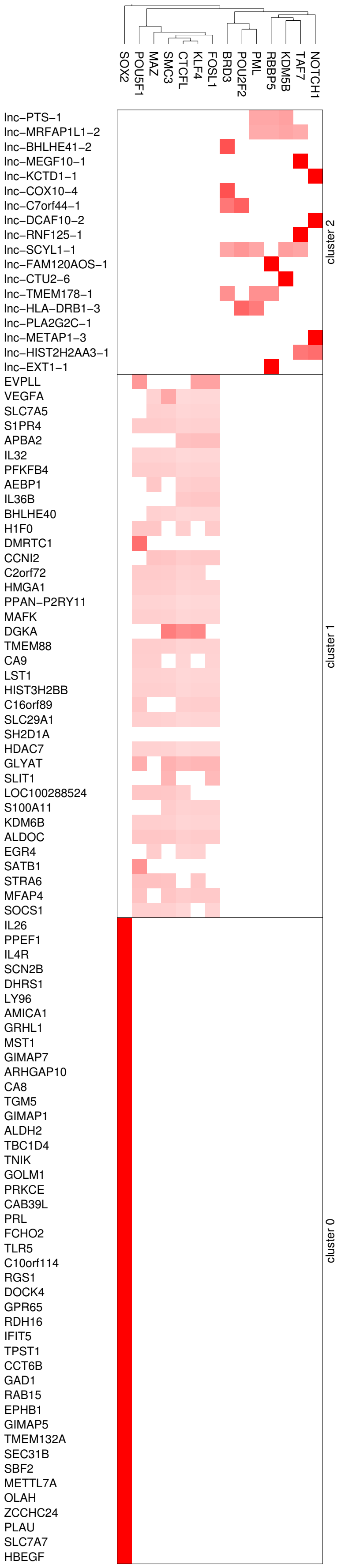

SOX2
POU5F1
MAZ
SMC3
CTCFL
KLF4
FOSL1
BRD3
POU2F2
PML
RBBP5
KDM5B
TAF7
NOTCH1
lnc−PTS−1
lnc−MRFAP1L1−2
lnc−BHLHE41−2
lnc−MEGF10−1
lnc−KCTD1−1
lnc−COX10−4
lnc−C7orf44−1
lnc−DCAF10−2
lnc−RNF125−1
lnc−SCYL1−1
lnc−FAM120AOS−1
lnc−CTU2−6
lnc−TMEM178−1
lnc−HLA−DRB1−3
lnc−PLA2G2C−1
lnc−METAP1−3
lnc−HIST2H2AA3−1
lnc−EXT1−1
EVPLL
VEGFA
SLC7A5
S1PR4
APBA2
IL32
PFKFB4
AEBP1
IL36B
BHLHE40
H1F0
DMRTC1
CCNI2
C2orf72
HMGA1
PPAN−P2RY11
MAFK
DGKA
TMEM88
CA9
LST1
HIST3H2BB
C16orf89
SLC29A1
SH2D1A
HDAC7
GLYAT
SLIT1
LOC100288524
S100A11
KDM6B
ALDOC
EGR4
SATB1
STRA6
MFAP4
SOCS1
IL26
PPEF1
IL4R
SCN2B
DHRS1
LY96
AMICA1
GRHL1
MST1
GIMAP7
ARHGAP10
CA8
TGM5
GIMAP1
ALDH2
TBC1D4
TNIK
GOLM1
PRKCE
CAB39L
PRL
FCHO2
TLR5
C10orf114
RGS1
DOCK4
GPR65
RDH16
IFIT5
TPST1
CCT6B
GAD1
RAB15
EPHB1
GIMAP5
TMEM132A
SEC31B
SBF2
METTL7A
OLAH
ZCCHC24
PLAU
SLC7A7
HBEGF
cluster 2
cluster 1
cluster 0

Supplemental Data 12. Enrichment analysis of HIV interactions of the lncRNA-mRNA neighboring pairs. (Fisher Exact test)

lncRNA strand	neighbor mRNA	category		array pairs	array hiv	de pairs	de hiv				
sense	up sense	1	TH	5464	419	91	13				
	up as	3	HH (nearby head)	4855	480	91	10				
	down sense	2	TH	644	416	81	8				
	down as	4	TT (nearby tail)	4916	412	92	8				
antisense	up sense	1	TH	4694	443	98	10				
	up as	3	HH (nearby head)	5365	373	101	10				
	down sense	2	TH	4739	522	100	19				
	down as	4	TT (nearby tail)	5391	434	98	10				
sense	overspanning lncRNA	5	full overlap	1823	318	29	3				
	intragenic of lncRNA	5	full overlap	4812	155	105	5				
	overlapping	6	partial overlap	2627	455	51	14				
antisense	overspanning lncRNA	5	full overlap	1832	321	25	5				
	intragenic of lncRNA	5	full overlap	5102	162	125	6				
	overlapping	6	partial overlap	2669	448	75	17				
								**p value**	**OR**	**OR low**	**OR high**
**TOTAL**				54933	5358	1162	138	0.019	1.25	1.03	1.5

Supplementary Data 13. Expression profiles of the 23 differentially expressed lncRNA-mRNA pairs upon HIV infection

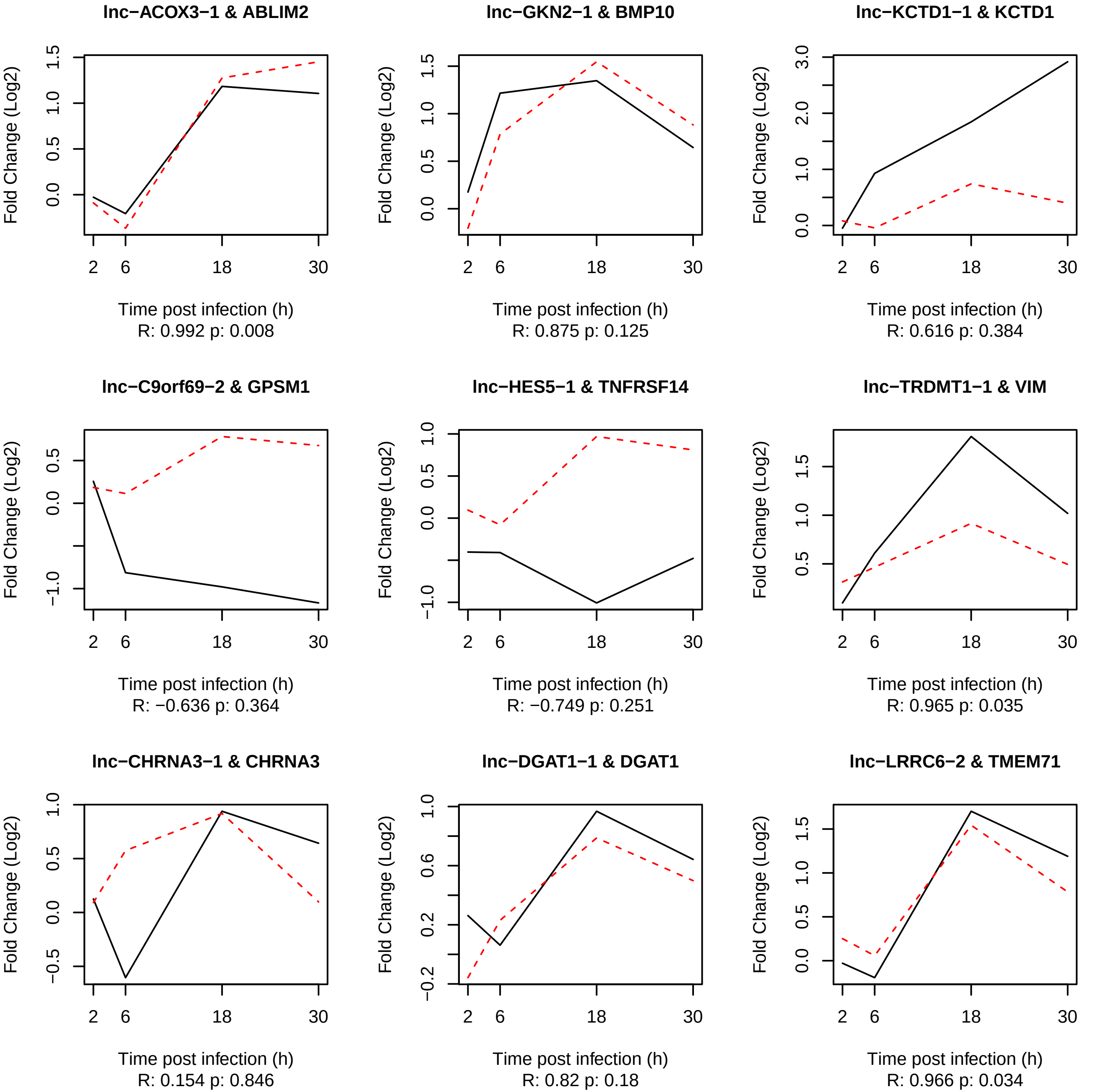
lnc−ACOX3−1 & ABLIM2
Fold Change (Log2)
Time post infection (h)
R: 0.992 p: 0.008
lnc−GKN2−1 & BMP10
Fold Change (Log2)
Time post infection (h)
R: 0.875 p: 0.125
lnc−KCTD1−1 & KCTD1
Fold Change (Log2)
Time post infection (h)
R: 0.616 p: 0.384
lnc−C9orf69−2 & GPSM1
Fold Change (Log2)
Time post infection (h)
R: −0.636 p: 0.364
lnc−HES5−1 & TNFRSF14
Fold Change (Log2)
Time post infection (h)
R: −0.749 p: 0.251
lnc−TRDMT1−1 & VIM
Fold Change (Log2)
Time post infection (h)
R: 0.965 p: 0.035
lnc−CHRNA3−1 & CHRNA3
Fold Change (Log2)
Time post infection (h)
R: 0.154 p: 0.846
lnc−DGAT1−1 & DGAT1
Fold Change (Log2)
Time post infection (h)
R: 0.82 p: 0.18
lnc−LRRC6−2 & TMEM71
Fold Change (Log2)
Time post infection (h)
R: 0.966 p: 0.034

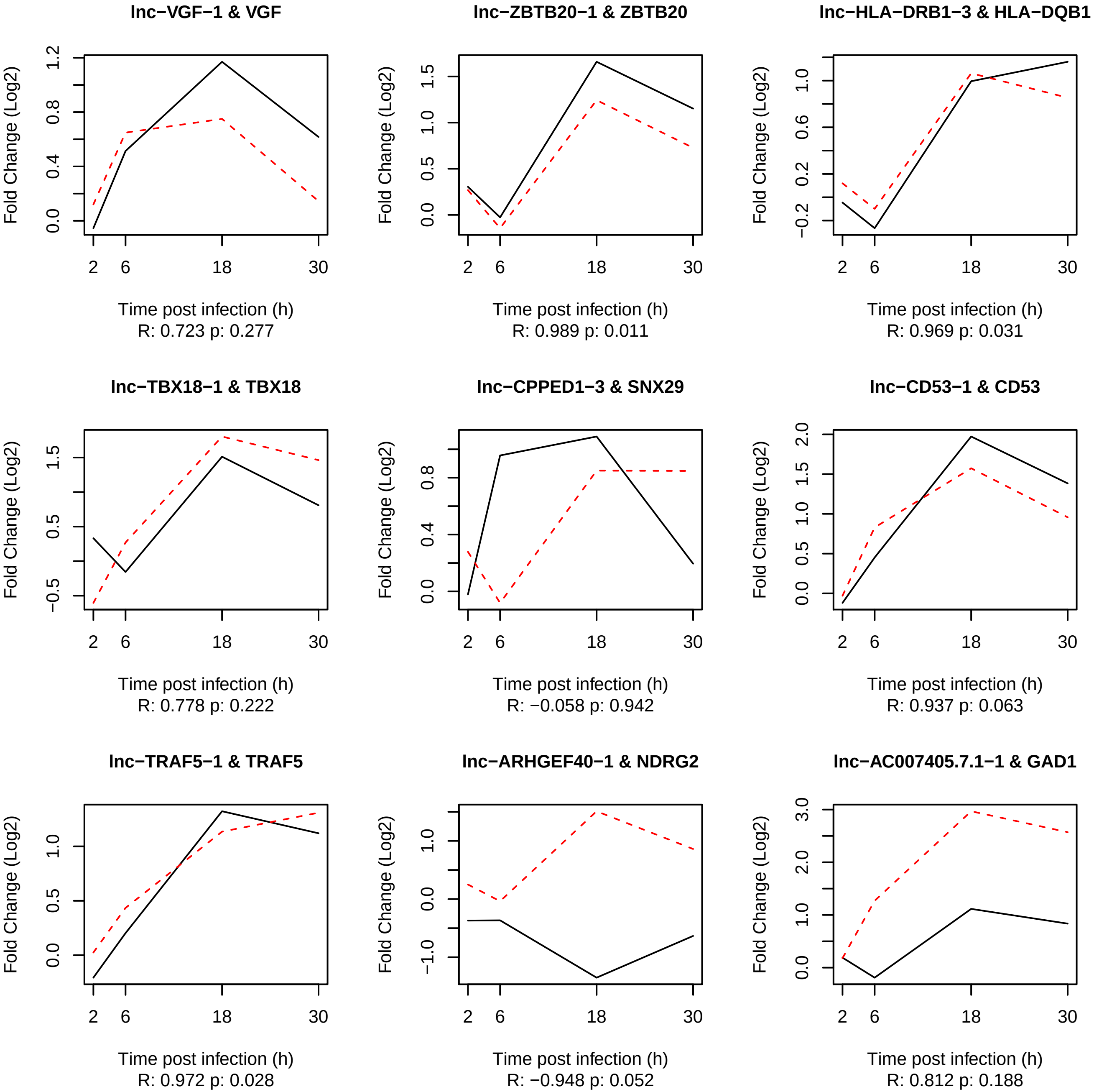
lnc−VGF−1 & VGF
Fold Change (Log2)
Time post infection (h)
R: 0.723 p: 0.277
lnc−ZBTB20−1 & ZBTB20
Fold Change (Log2)
Time post infection (h)
R: 0.989 p: 0.011
lnc−HLA−DRB1−3 & HLA−DQB1
Fold Change (Log2)
Time post infection (h)
R: 0.969 p: 0.031
lnc−TBX18−1 & TBX18
Fold Change (Log2)
Time post infection (h)
R: 0.778 p: 0.222
lnc−CPPED1−3 & SNX29
Fold Change (Log2)
Time post infection (h)
R: −0.058 p: 0.942
lnc−CD53−1 & CD53
Fold Change (Log2)
Time post infection (h)
R: 0.937 p: 0.063
lnc−TRAF5−1 & TRAF5
Fold Change (Log2)
Time post infection (h)
R: 0.972 p: 0.028
lnc−ARHGEF40−1 & NDRG2
Fold Change (Log2)
Time post infection (h)
R: −0.948 p: 0.052
lnc−AC007405.7.1−1 & GAD1
Fold Change (Log2)
Time post infection (h)
R: 0.812 p: 0.188

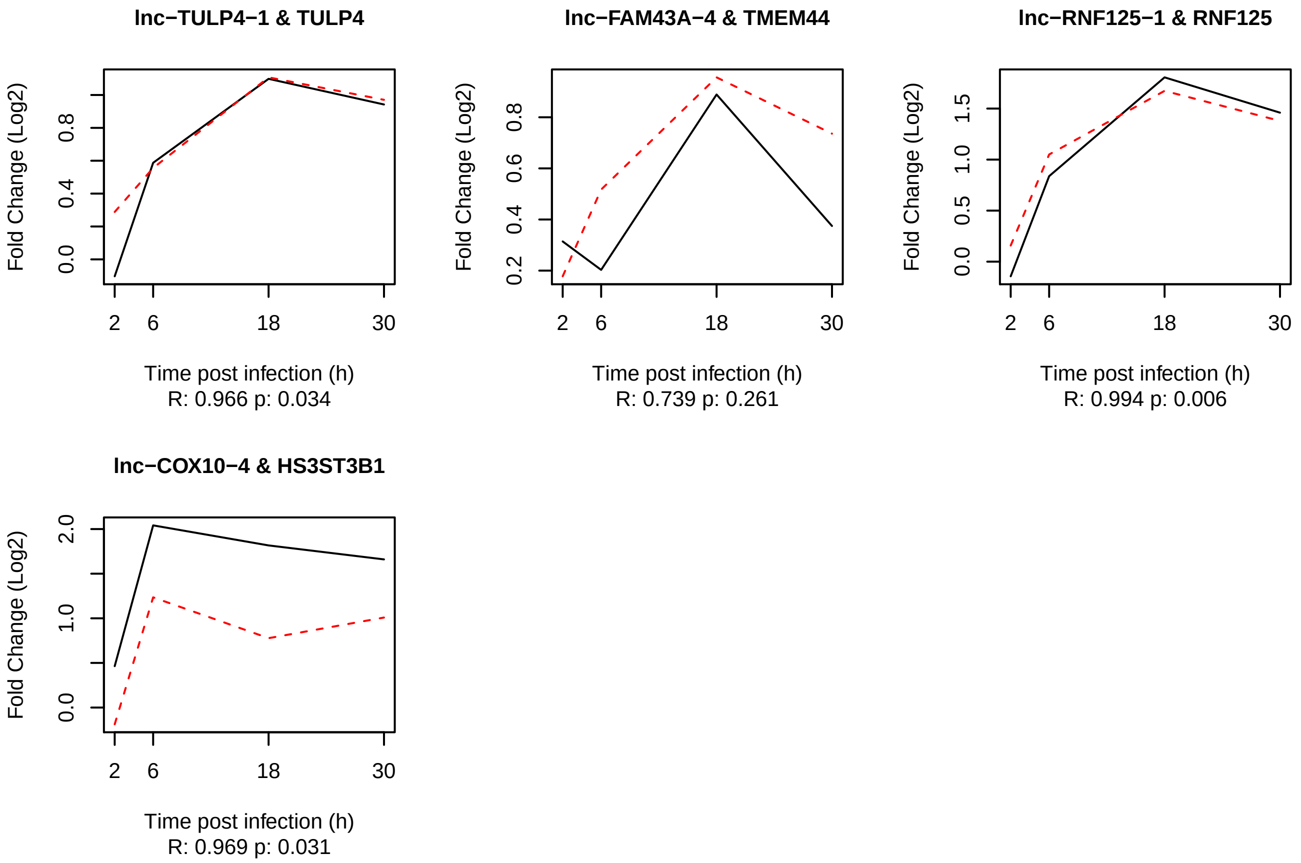
lnc−TULP4−1 & TULP4
Fold Change (Log2)
0.0
0.4
0.8
2
6
18
30
Time post infection (h)
R: 0.966 p: 0.034
lnc−FAM43A−4 & TMEM44
Fold Change (Log2)
0.2
0.4
0.6
0.8
2
6
18
30
Time post infection (h)
R: 0.739 p: 0.261
lnc−RNF125−1 & RNF125
Fold Change (Log2)
0.0
0.5
1.0
1.5
2
6
18
30
Time post infection (h)
R: 0.994 p: 0.006
lnc−COX10−4 & HS3ST3B1
Fold Change (Log2)
0.0
1.0
2.0
2
6
18
30
Time post infection (h)
R: 0.969 p: 0.031

Supplementary Data 14. Epienetic context of the 5 HIV linked mRNA-lncRNA pairs. Visualization by IGV.

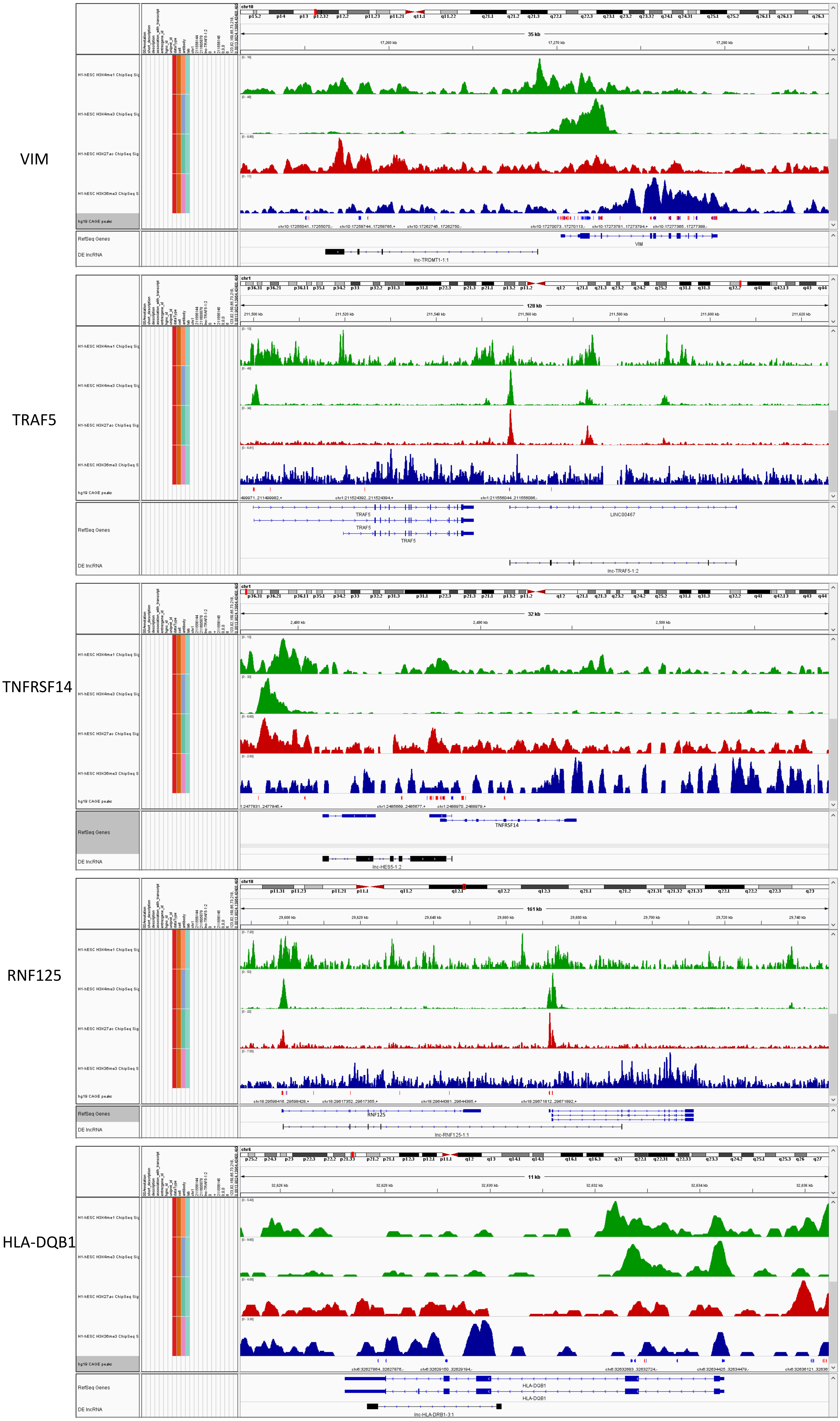

Supplementary Data 15. Co-expression in other datasets of 3/5 HIV lncRNA-mRNA pairs. Co-LncRNA Blood datasets: Pickrell et al. and Kasowki et al.

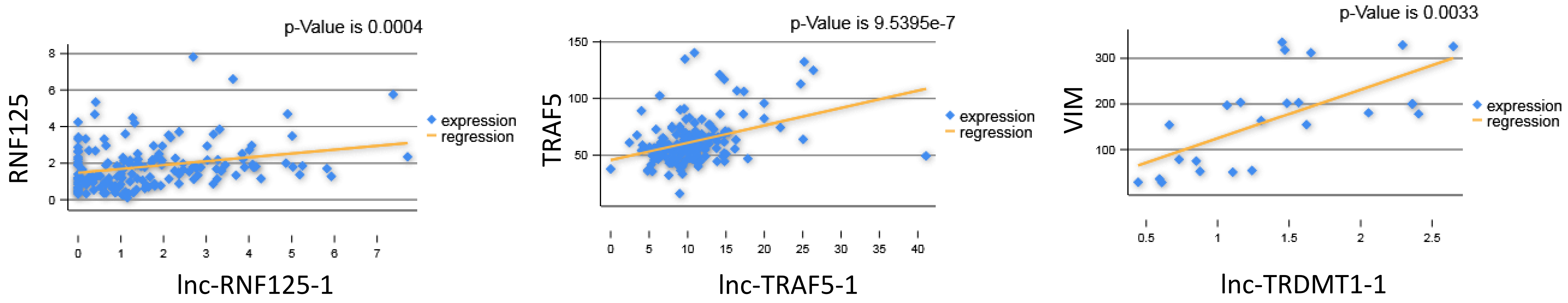

Supplement: Supplementary Information [file srep36111-s1.pdf]
